# Diffusion of Sodium Hyaluronate in Artificial Saliva to Optimize Its Topical Application

**DOI:** 10.3390/molecules30102140

**Published:** 2025-05-13

**Authors:** Francisco J. R. Carmo, Esmeraldo P. Z. Salote, Artur J. M. Valente, Ana C. F. Ribeiro, Pedro M. G. Nicolau, Sónia I. G. Fangaia

**Affiliations:** 1 Faculty of Medicine, University of Coimbra, Av. Bissaya Barreto, Blocos de Celas, 3000-075 Coimbra, Portugal; franciscoramalhocsrmo@gmail.com (F.J.R.C.); pnicolau@fmed.uc.pt (P.M.G.N.); 2 CQC-IMS, Department of Chemistry, University of Coimbra, 3004-535 Coimbra, Portugal; esmeraldosalote@gmail.com (E.P.Z.S.); avalente@ci.uc.pt (A.J.M.V.); 3 CIROS, Center for Innovation and Research in Oral Sciences, Institute of Implantology and Prosthodontics, University of Coimbra, Av. Bissaya Barreto, Blocos de Celas, 3000-075 Coimbra, Portugal; 4 Center of Mechanical Engineering Materials and Processes (CEMMPRE), Department of Engineering Mecânica Pinhal de Marrocos, University of Coimbra, 3030-788 Coimbra, Portugal

**Keywords:** hyaluronic acid, mouth rinse, mutual diffusion, oral mucous regeneration, topical application

## Abstract

Hyaluronic acid (or hyaluronan) is a polysaccharide with therapeutic applications in dentistry due to its lubricating, anti-inflammatory, and antibacterial properties. This study evaluates the diffusion, conductivity, and viscosity of the sodium salt of HyH (that is, NaHy) with different molecular weights (124 kDa, 245 kDa, and 1800 kDa) in artificial saliva at pH 2.3, 4, 5, 6.8, and 8. Using the Taylor dispersion technique at 298.15 K, diffusion coefficients were determined and analyzed based on Fick’s second law equation. Results showed that NaHy diffusion was higher at acidic pH, particularly at pH 2.3, and decreased at pH 8, likely due to structural compaction in acidic conditions and expansion in alkaline media. The higher molecular weight of this polysaccharide exhibited greater diffusion and conductivity, suggesting an extended conformation that enhances mobility. These findings indicate that both pH and molecular weight significantly influence NaHy transport properties. Optimizing these parameters may enhance HA’s bioavailability and effectiveness in topical oral applications, improving its therapeutic potential in treating periodontal and oral conditions.

## 1. Introduction

Nowadays, hyaluronic acid (HyH) has been widely studied not only in the cosmetic industry but increasingly in the medical context as a treatment method for dermatological, ophthalmological, and orthopedic issues and even as an ally in dental medicine [1,2]. However, despite the growing number of publications in this area, they have proven to be insufficient due to their recent nature and the lack of long-term experimental results.

Hyaluronic acid (HyH, C_14_H_21_NaNO_11_)n is a polysaccharide belonging to the family of non-sulfated glycosaminoglycans and is the one with the highest molecular weight (4000–20,000,000 Da). The structure of hyaluronic acid (HyH) consists of polyanionic disaccharide units of glucuronic acid and *N*-acetylglucosamine connected by alternating β1-3 and β1-4 glycosidic bonds, respectively [3,4].

This substance is present in all vertebrate tissues, playing a vital role in the functioning of the extracellular matrix of both mineralized and non-mineralized tissues. It can be found in high concentrations in the skin, synovial fluid, and periodontal structures, specifically in the gingiva, periodontal ligament, and crevicular fluid. It is also present in mineralized structures such as alveolar bone and cementum, although in lower concentrations, and is found in trace amounts in blood plasma [5,6,7]. However, hyaluronic acid is very poorly soluble in water unless it is converted to the ionized form, giving rise to hyaluronate, which is water-soluble. For this reason, it is common to find studies in the literature involving the sodium salt of hyaluronic acid (NaHy), targeting different applications (e.g., medical and cosmetic) [8,9,10,11].

This salt can be derived from either animal sources or semi-synthetic processes. Its therapeutic applications are widespread due to its non-toxic, biocompatible properties. The semi-synthetic variant is often preferred because it allows for large-scale production and minimizes allergic reactions, as it does not involve the incorporation of animal proteins, unlike HyH derived from animal sources [8,9].

Because of its high molecular weight and special viscoelastic features [12], hyaluronic acid exhibits properties of lubrication, tissue repair, anti-inflammatory, anti-edematous, and antibacterial effects and is one of the most hygroscopic molecules known in nature [13,14,15]. For these reasons, it has been clinically applied in medicine, particularly in dentistry, for the treatment of numerous oral and periodontal conditions (HyH has comparable roles in the healing of the mineralized and nonmineralized tissues). It is used in managing chronic inflammatory diseases, such as gingival inflammation, or as an adjunct in non-surgical periodontal therapy for periodontitis [15,16]. In the treatment of temporomandibular disorders, HyH is increasingly used due to being effective in the treatment of inflammatory and biomechanical alterations in the temporomandibular joint; a supplementation of joint fluids alleviates pain and improves mobility in individuals with conditions such as osteoarthritis, where synovial fluid, the natural joint lubricant, becomes less effective [2,4,17,18,19].

**Figure 1:**
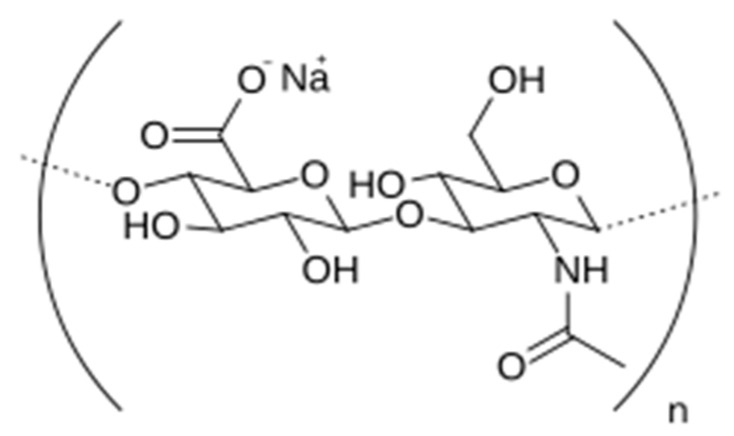
Structure of the disaccharide repeating unit in sodium hyaluronate.

In this work we are particularly interested in the use of sodium salt of hyaluronic acid (NaHy) (Figure 1) in the form of mouth rinses for the treatment and prevention of traumatic ulcers, caused by removable dental prostheses, in totally and partially edentulous patients. As is known, these patients, often elderly, have a considerable decrease in salivary flow due to various causes, namely systemic diseases such as diabetes and Sjogren’s syndrome, polymedication, and atrophy of the salivary glands, for example [20]. Removable dentures, as they are in direct contact with the oral mucosa, cause friction, which, in the absence of the lubricating effect of saliva, leads to the appearance of traumatic ulcers, which are almost always painful and have a major impact on the well-being of patients, especially as they affect their diet [20,21,22]. Therefore, as far as we are concerned, these patients can benefit from the hydrating, lubricating, and anti-inflammatory properties of NaHy on their oral mucosa [23,24]. Using a mouth rinse formulation with HyH offers distinct advantages: not only is it better accepted by patients, but it also allows treatment to reach areas of the oral cavity that might be inaccessible through topical application in gel formulations [25,26], and topical treatments seem to be more effective in their ability to deliver high concentrations of pharmacological agents to the teeth and oral mucosa than systemic administration [3].

However, the understanding of the systems composing NaHy is not yet well established, making its characterization fundamental. This study assists in elucidating its structure and developing methods for practical applications in dental medicine. Despite the increasing number of studies in this field, few consider its transport behavior [27,28]. Transport properties, especially the mutual diffusion coefficients of this polysaccharide (also referred to as inter-diffusion coefficients), which involve coupled fluxes of solutes and solvent molecules driven by concentration gradients, provide a direct measure of molecular mobility—an essential factor in the preservation of biological materials in physiological solutions [29]. This study aims to investigate the behavior of mutual diffusion of NaHy of different molecular weights at therapeutic concentrations (≤0.1% (*m*/*v*), the usual concentration of NaHy in mouth rinse formulations [26,27,30,31]), specifically in solutions with varying pH levels. Despite a thorough literature review, no data were found regarding mutual diffusion coefficients in physiological solutions containing this polysaccharide, particularly concerning its topical application [31].

## 2. Results

### 2.1. Diffusion Measurements

Table 1 summarizes the mean values and the respective standard deviations of the diffusion coefficients for water and solutions of different compositions for five physiological media, involving the artificial saliva and sodium hyaluronate (NaHy) with three different molecular weights at different pH values (pH measurements were taken for all the solutions containing 0.01 g of NaHy and 10 mL of artificial saliva). It should be noted that ultrapure water, ρ = 1.82 × 10^5^ Ω m at 298.15 K, was used in measurements of diffusion coefficients, conductivity, and viscosity, and that artificial saliva was prepared according to the composition indicated in Section 4, and using also this water. The values were calculated by fitting Equation (1) to dispersion curves. The standard deviations remain relatively low across the measurements (Table 1), showing an acceptable uncertainty of <3%, typical for Taylor dispersion measurements.

From the analysis of Table 1, similar behaviors can be seen in the diffusion of NaHy with different molecular weights (124 kDa, 245 kDa, and 1800 kDa) in water and in artificial saliva at different pH values. That is, the diffusion coefficients of NaHy in these media tend to decrease as the pH increases. The highest diffusion coefficients are recorded at lower pH values (e.g., pH = 2.3, and for NaHy with Mw = 124 kDa, D = from 0.960 × 10⁻⁹ m^2^/s), and a sharp reduction is observed as the pH increases to 8.0 (e.g., pH = 8.0, and for NaHy with the same MW, D = from 0.560 × 10⁻⁹ m^2^/s).

### 2.2. Conductivity Measurements

**Figure 2:**
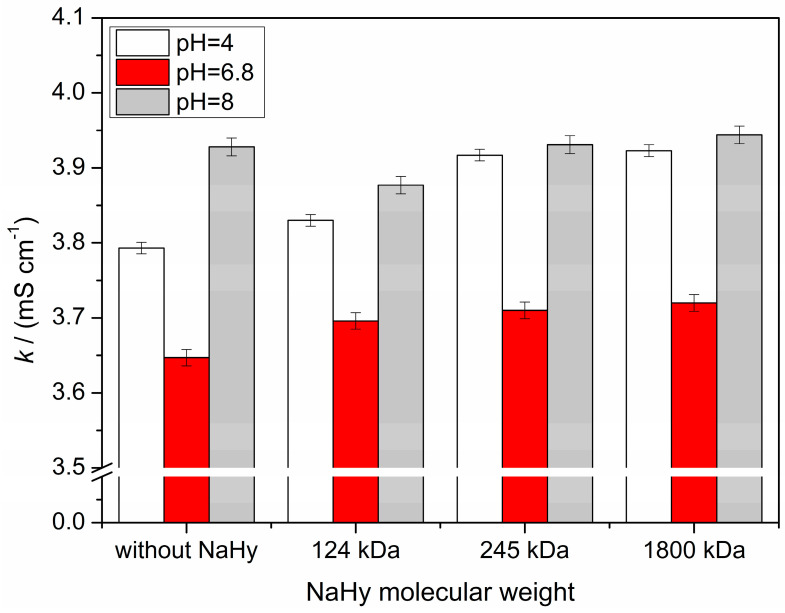
Conductivity of artificial saliva at 298.15 K and at different pHs (4, 6.8, and 8) with and without NaHy 0.1% (*m*/*v*) with different molecular weights (Mw (NaHy) = 124 kDa; Mw (NaHy) = 245 kDa; Mw (NaHy) = 1800 kDa).

Figure 2 shows the values of conductivity of artificial saliva-based solutions at different pH values in the absence and presence of NaHy.

The data from Figure 2 present the electrical conductivity of samples of artificial saliva without and with varying molecular weights (124 kDa, 245 kDa, and 1800 kDa) under three different pH conditions (4, 6.8, and 8). The results indicate a notable conductivity variation depending on pH and molecular weight.

At pH 4, the conductivity values range from 3.793 mS/cm (in the absence of NaHy) to 3.923 mS/cm (NaHy of Mw = 1800 kDa).

The lowest conductivity values are recorded at pH 6.8, ranging from 3.696 mS/cm (124 kDa) to 3.719 mS/cm (1800 kDa).

At pH 8 the conductivity values are significantly higher compared to pH 6.8 (<24%) and slightly higher than pH 4.

The conductivity decreases for the NaHy of Mw = 124 kDa fraction but slightly increases as molecular weight increases.

### 2.3. Density and Viscosity Measurements

The density and viscosity values for nine systems were the means of at least ten sets of measurements (Table 2).

From this table, we can infer that at each pH considered, there were no significant differences between the densities of the solutions of NaHy with differing molecular weights (≤1%). The lower values are found for acidic solutions, probably due to structural compaction in these conditions.

Relative to the viscosity data, we can observe that, in general, this property of solutions increases with the increase in the pH. Without sodium hyaluronate, the viscosity of artificial saliva is lower and remains almost the same value across all pHs (deviations < 2%).

Relative to the samples of NaHy (Mw = 124 kDA, and 1800 kDa), the variations in viscosity between them are small overall at pH 6.8 (5%).

However, at pH 4 and 8, a higher molecular weight (1800 kDa) leads to higher viscosity compared to pH 6.8.

The low molecular weight was not significantly relevant to the viscosity values regardless of pH.

## 3. Discussion

From analysis of Table 1, it can be observed that there is a dependence of the diffusion behavior of NaHy on the pH of the saliva and water, regardless of its molecular weight. In other words, the diffusion coefficient of NaHy decreases drastically when passing from pH 2.3 to pH 8 (e.g., for solutions containing NaHy with a molecular weight of 124 kDa, D(NaHy) decreases to 40% in this variation in pH). In contrast, the conductivity of these solutions slightly increases when the pH value varies from 4 to 8.

Using an Onsager–Fuoss model [32,33], we can assume that two different effects can control the diffusion process: the ionic mobility and the gradient of the chemical potential. Based on these measurements, in connection with the conductance measurements (Figure 2), assuming that *D* is a product of both kinetic (*F*_M_) and thermodynamic factors (*F*_T_) [33], we suggest that the thermodynamic factor decreases and the kinetic factor increases when the saliva pH increases from 4 to 8, with both systems having this polysaccharide.

In mathematical terms,

*D* = *F_M_* × *F*_T_ (1)

*F*_M_ = U_m_ (2)

(3) $$F_{T}=\left(1+c\frac{\partial \mu }{\partial c}\right)$$

where U_m_ and μ represent the molar mobility coefficient of a diffusing substance and the chemical potential, respectively. However, it is worth noting that this model is only valid in the diluted region, where the change in the viscosity can be neglected [33].

In the present work, for saliva at pH 8 containing NaHy of molecular weight 124 kDa, the viscosity assumes higher values (Table 2) and should be considered in the analysis of the mass transport by diffusion. It can be found that the increase in viscosity observed for this system has a main role in the decrease in the diffusion coefficient of NaHy [33]. In this case, the thermodynamic factor can be corrected, $F_{T}^{\prime }$, and consequently, the effect of pH becomes more significant.

In short, it can be concluded that the decrease in diffusion coefficients can arise from increasing the mobility of NaHy, decreasing the gradient of the chemical potential with concentration, and increasing the viscosity of artificial saliva in the presence of NaHy.

From similar values of conductivity (*κ*) (or *F*_M_) (Figure 2) for the pH range, we can say that the pH has a small effect on *F*_M_. In other words, due to the similar conductivity behavior of NaHy observed at this pH, the changes in the mobility factor with concentration do not seem to be as relevant as when compared to those obtained for the thermodynamic factor. From these findings, we can conclude that the variation in *D* is mainly due to the variation in $F_{T}^{\prime }$ (attributed to the non-ideality in thermodynamic behavior) and to the variation in viscosity.

(4) $$F_{T}^{\prime }=F_{T}\frac{\eta_{r}}{\eta }$$

and

(5) $$\eta_{r}=\frac{\eta }{\eta_{w}}$$

where *η* and *η_w_* represent the viscosity of the solution and water.

In fact, considering viscosity data (Table 2), the corresponding $F_{T}^{\prime }$ values are higher than those of *F*_T_ calculated from Equation (4). This increase in the thermodynamic factor makes the contribution of the *F*_M_ factor even smaller when the molecular weight of NaHy becomes higher.

One possible explanation for these findings may be explained on the basis of the perturbation of the H-bond network caused by increasing pH. That is, the -OH groups’ dissociation can reduce the number of H bonds between -OH and different groups, such as the carboxylic acid of lactic acid or acetamido groups, which control the local stiffness of the HA molecule. The partial rupture of the H-bond network and the increase in the polymer net charge, as well as the increase in other ionic species (e.g., lactate anion), can be responsible for the increase in the frictional resistance, which acts towards the decrease in the diffusion coefficient (unlike electric conductivity, which is increased by dissociation of this network). Relative to the viscoelastic properties of NaHy for two different molecular weights over a pH range, it was observed that the variation in the viscosity of saliva at pH values 4 and 8 containing NaHy of different molecular weights is relatively small (that is, 6% and 24% for 124 kDa and 1800 kDa). This may indicate that within the pH range studied, there is no degradation of this polymer, despite the possibility of some disruption of H-bonds. Support for these facts comes from a recent rheological investigation on the effect of pH in semi-dilute solutions, where the disruption of the HA chains ascribed to cleavage of glycosidic bonds occurs more probably at low and high pH (pH 1 and 12) [34]. Furthermore, if degradation were to occur, the viscosity of these media should decrease. This decrease is mainly attributed to the reduction in the rigidity of the polymer structure under alkaline conditions due to the partial breaking of the hydrogen bond network [35]. However, in our work, we observed the opposite situation when obtaining higher values for this parameter for saliva with NaHy of lower molecular weight at pH 8 (Table 2), which can indicate the eventual presence of polymer–polymer interactions more accentuated in this case [36].

In summary, contrary to the strong influence of pH on the transport behavior of NaHy, the molecular weight of NaHy does not significantly influence the viscosity and conductivity of these specific systems; however, the same does not apply to diffusion or other types of systems [27]. For example, in this last work, the authors, when studying the effect of the molecular weight of NaHy on viscosity and lubrication, concluded that NaHy with lower molecular weight presented better wettability than the other NaHy samples.

It should be noted that no attempt is made at this stage to separate the individual contributions relating to the interactions of the species involved that explain the behavior of the transport properties analyzed in the present work (*D*, *k,* and *η*), since what is needed in these practical applications, i.e., the chemistry of the oral cavity, is knowledge of global behavior.

According to literature, NaHy plays a multifunctional role in wound healing and soft tissue repair. The data obtained in this study are of fundamental importance for obtaining topical pharmacological formulations that are more effective on damaged oral mucosa, also benefiting from its action type, like the cushioning and lubrication of several tissues, including the soft tissues of the oral mucosa.

Our results indicate that a formulation that undergoes less diffusion and that consequently remains in contact with the tissues for longer must be made up of a low molecular weight HA in a matrix with a basic pH.

## 4. Materials and Methods

### 4.1. Materials

Table 3 describes all the reagents received in the present work, including sodium hyaluronate with different molecular weights (124 kDa, 245 kDa, and 1800 kDa) and artificial saliva (8 pH). All chemicals were used without further purification. The solutions needed for diffusion measurements were prepared in calibrated volumetric glass flasks, using artificial saliva with different pHs as solvent (artificial saliva was prepared according to the composition indicated in Table 3, and using ultrapure water ρ = 1.82 × 10^5^ Ω m at 298.15 K). The weighing was performed using a Radwag AS 220C2 balance (Radwag, Radom, Poland), with an accuracy of ±0.0001 g.

### 4.2. Experimental Techniques

#### 4.2.1. pH Measurements

The pH measurements of solutions were carried out with a Radiometer pH meter PHM 240 with an Ingold U457-K7 pH conjugated electrode; pH was measured in fresh solutions, and the electrode was calibrated immediately before each experimental set of solutions using IUPAC-recommended pH 4, 7, and 10 buffers. From pH meter calibration, a zero pH of 6.897 ± 0.030 and sensitivity higher than 98.7% were obtained. To perform these measurements at pH 2.0, 3.0, 4.9, 5.0, 6.8, and 8.0, the pH’s intended values were adjusted by adding lactic acid. Before each experiment, each solution was vibrated and degassed by sonication before each experiment.

#### 4.2.2. Taylor Dispersion Technique

The Taylor diffusion technique allows the measurement of diffusion coefficients in multicomponent systems and, as the name implies, is based on the work carried out by G.I. Taylor in the 1950s of the last century, being profusely described in the literature [33,37,38,39,40].

A summary of the most relevant issues related to the technique will be described in the following section. As a common feature of all chromatographic-based techniques, a disperse profile is obtained by injecting a volume equal to 0.063 mL of solution, at the beginning of the experiment, into a Teflon tube with a length and internal diameter of 3048.0 (±0.1) cm and 0.06440 ± (0.00006) cm, respectively, where a solution of defined concentration and composition flows in laminar flow. All equipment is thermostated at a temperature of 298.15 (±0.01) K. The dispersion obtained in the sequence of different flows of the different species is registered using a differential refractometer (Waters model 2410). This equipment measures the electric potential as a function of time, *V*(*t*), by coupling a digital voltmeter (Agilent 34,401 A) at the outlet of the dispersion tube. Binary diffusion coefficients were evaluated by fitting Equation (6) to the obtained dispersion profile [38].

(6) $$V\left(t\right)=V_{0}+V_{1}t+{V_{max}(tR/t)}^{1/2}\;exp[-12D{\left(t-t_{R}\right)}^{2}/{r\;}^{2}t]$$

In Equation (6), the additional fitting parameters were the mean sample retention time $t_{R}$, peak height $V_{max}$, baseline voltage $V_{0}$, and baseline slope $V_{1}$.

These systems can be considered binary due to the fact that the concentration of the artificial saliva (component 1) is significantly higher than the concentration of HA (component 2). You can also find more information on how the binary diffusion coefficients can be calculated in references [28,41,42].

#### 4.2.3. Conductivity

The electrical resistances of NaHy solutions were measured with an automatic LCR meter (model 4265, Wayne-Kerr) at 1 kHz equipped with a Shedlovsky-type conductance cell. The cell constant value of 0.1181 ± 0.0002 cm^−1^ was obtained from conductivity measurements of dilute aqueous potassium chloride solutions according to [43].

In a typical experiment, 20 mL of artificial saliva solution with and without three different molecular weight concentrations was placed in the conductivity cell. Upon achieving homogenization after each addition of NaHy, the electrical resistance of the resulting solution is measured. This procedure is consecutively repeated for each one of the concentrations studied. In all cases, the electrical resistance of every solution is measured three times, and their average value is used to calculate conductance. All experiments were performed at 268.15 (±0.01) K in a Grant thermostat bath. Solutions were always used within 12 h of preparation. The estimated uncertainty for these electrical conductance values is less than 0.1%.

#### 4.2.4. Density

The density of these solutions was determined with an Anton Paar DMA5000M densimeter (precision of 1 × 10^−6^ g·cm^−3^ and accuracy of 5 × 10^−6^ g·cm^−3^ in the ranges of (0–90) °C of temperature and (0–1.0) MPa of pressure). The measurement uncertainty for density was estimated to be 0.001%.

#### 4.2.5. Viscosity

The relative viscosity measurements of the prepared solutions were conducted using an Ubbelohde-type suspended-level viscometer. To ensure thermal stability, the viscometer containing the test solution was maintained in a thermostatic water bath for approximately 45 min, minimizing temperature fluctuations. Calibration of the viscometer was performed with deionized distilled water over a temperature range of 298.15–313.15 K. The efflux time for each test solution was recorded three times using a digital stopwatch with a precision of ±0.01 s, and the mean of these readings was utilized for viscosity calculations. The measurement uncertainty for viscosity was estimated to be 0.01%.

## 5. Conclusions

Hyaluronan is a versatile and multifaceted macromolecule that applied topically, provides added value in tissue regeneration. By assessing the diffusion, conductivity, and viscosity of hyaluronic acid of different molecular weights and at different pH, the results indicate that the behavior of hyaluronic acid is strongly influenced by the pH. Additionally, it was observed that the molecular weight of NaHy does not have a significant impact on conductivity as opposed to diffusion and viscosity. Therefore, it can be inferred that the most suitable conditions for reduced diffusion, and consequently, greater topical efficiency of hyaluronic acid, are preferably in an alkaline matrix, especially when associated with a low molecular weight.

Additional studies are needed, such as extending this work to other temperature ranges or to other salts of hyaluronate or using simulated salivary fluid containing proteins; meanwhile, the research reported and discussed in this manuscript is useful for allowing the understanding of the main features of this complex system at the standard temperature of 25 °C, including the composition dependence of the diffusion coefficients, densities, and viscosities at different pH values.

This knowledge is essential for the development of hyaluronic acid-based formulations for oral care, particularly in the form of mouthwashes for dental medicine, which can be significantly optimized by careful control of key parameters. The use of low molecular weight hyaluronic acid within an alkaline matrix was shown to minimize diffusion, thereby ensuring prolonged contact with the oral mucosa and maximizing its therapeutic effects. This prolonged retention enhances HA’s natural lubricating, cushioning, and regenerative properties, contributing to faster and more efficient soft tissue repair. Therefore, mouthwash formulations tailored with a basic pH environment and appropriately selected HA molecular weights present a promising strategy for improving the treatment of oral mucosal injuries, surgical wounds, and inflammatory conditions, ultimately elevating patient outcomes and advancing oral healthcare practices, with increased retention time and therapeutic potential in biomedical and pharmaceutical applications.

## Figures and Tables

**Table 1:** pH and diffusion coefficient measurements (*D*) for water and artificial saliva with and without sodium hyaluronate with three different molecular weights at 298.15 K.

Solutions	Solutions at Different pHs	*D* ± *S_D_* ^a)^ /(10^−9^m^2^ s^−1^)
Sodium hyaluronate (Mw = 124 kDa) 0.01 g in 10 mL (0.1%) + Water	4.0 6.8 8.0	0.815 ± 0.010 0.698 ± 0.007 0.560 ± 0.003
Sodium hyaluronate (Mw = 245 kDa) 0.01 g in 10 mL (0.1%) + Water	4.0 6.8 8.0	0.880 ± 0.010 0.708 ± 0.007 0.580 ± 0.003
Sodium hyaluronate (Mw = 1.8 MDa) 0.01 g in 10 mL (0.1%) + Water	4.0 6.8 8.0	0.970 ± 0.010 0.880 ± 0.014 0.770 ± 0.021
Sodium hyaluronate (Mw = 124 kDa) 0.01 g in 10 mL (0.1%) + Artificial Saliva	2.3 4.0 5.0 6.8 8.0	0.960 ± 0.021 0.862 ± 0.018 0.810 ± 0.014 0.696 ±0.012 0.562 ± 0.013
Sodium hyaluronate (Mw = 245 kDa) 0.01 g in 10 mL (0.1%) + Artificial Saliva	2.3 4.0 5.0 6.8 8.0	0.964 ± 0.016 0.880 ± 0.028 0.850 ± 0.024 0.710 ± 0.021 0.611 ± 0.025
Sodium hyaluronate (Mw = 1800 kDa) 0.01 g in 10 mL (0.1%) + Artificial Saliva	2.3 4.0 5.0 6.8 8.0	0.997 ± 0.029 0.990 ± 0.030 0.870 ± 0.025 0.811 ± 0.027 0.780 ± 0.020

^a)^ *D* ± *S*_D_/(10^−9^ m^2^ s^−1^) represents the mean diffusion coefficients of six replicate measurements and the corresponding standard deviation of the mean. These data were obtained by injecting artificial saliva containing NaHy at different molecular weights into carrier artificial saliva at different pH.

**Table 2:** Density (ρ) and viscosity (*η*) measurements of samples of artificial saliva ^(a)^ without and with sodium hyaluronate of different molecular weights (NaHy 124 kDa and 1800 kDa).

Solution	$\rho /(g\;{cm}^{-3})\;$ ^(b)^	10^6^ σ ^(c)^	*η*/(mPa s) ^(d)^	10^3^ σ ^(e)^
Saliva (pH 8)	1.007105	1.6	0.9101	0.9
Saliva + NaHy (124 kDa) pH 8	1.002205	2.2	4.1101	0.9
Saliva + NaHy (1800 kDa) pH 8	1.016001	1.8	1.0800	0.8
Saliva (pH 6.8)	1.006911	2.8	0.9102	1.2
Saliva + NaHy (1800 kDa) pH 6.8	0.978590	2.9	1.2510	0.9
Saliva + NaHy (124 kDa) pH 6.8	0.979500	2.5	1.2090	1.1
Saliva (pH 4)	0.984423	2.7	0.9100	1.0
Saliva + NaHy (124 kDa) pH 4	0.983599	3.0	3.2010	0.7
Saliva + NaHy (1800 kDa) pH 4	0.985598	2.8	1.1701	0.8

^(a)^ Artificial saliva was prepared according to the following composition: potassium chloride (KCl, 20 mmol/L), sodium hydrogenocarbonate (NaHCO_3_, 17.9 mmol/L), sodium phosphate (NaH_2_PO_4_.H_2_O, 3.6 mmol/L), potassium thiocyanate (KSCN, 5.1 mmol/L), and lactic acid (0.10 mmol/L) [28,31]. ^(b)^ ρ represents the mean of ten sets of density measurements for each system. ^(c)^ σ represents the standard deviation of all mean density measurements. ^(d)^ *η* represents the mean of ten sets of viscosity measurements for each system. ^(e)^ σ represents the standard deviation of all viscosity measurements.

**Table 3:** Description of materials.

Chemical Name	Source	CAS Number	Mass Fraction Purity
Sodium hyaluronate ^(a)^ (Mw = 124 kDa) Sodium hyaluronate ^(a)^ ((Mw = 245 kDa) Sodium hyaluronate ^(a)^ ((Mw = 1800 kDa)	Contipro Ltd. (Dolní Dobrouč, Czech Republic) Contipro Ltd. (Dolní Dobrouč, Czech Republic) Contipro Ltd. (Dolní Dobrouč, Czech Republic)	9067-32-7 9067-32-7 9067-32-7	>0.99 >0.99 >0.99
Artificial Saliva ^(b)^	Dentistry area at the University of Coimbra of the Faculty of Medicine	-	-
Millipore-Q water $(\rho =1.82\;\times \;10^{5}\;\Omega \;m\;\mathrm{and}\;\rho$ = 0.99710 g cm^−3^ at 298.15 K)	-	7732-18-5	>0.97

^(a)^ Values provided by the suppliers; ^(b)^ Artificial saliva was prepared according to the following composition: potassium chloride (KCl, 20 mmol/L), sodium hydrogenocarbonate (NaHCO_3_, 17.9 mmol/L), sodium phosphate (NaH_2_PO_4_.H_2_O, 3.6 mmol/L), potassium thiocyanate (KSCN, 5.1 mmol/L), and lactic acid (0.10 mmol/L) [30,32].

## Data Availability

All data are presented in this paper.
